# Two types of laminolysis in adolescent athletes

**DOI:** 10.1007/s10195-012-0206-y

**Published:** 2012-07-20

**Authors:** Ryo Miyagi, Koichi Sairyo, Toshinori Sakai, Hiroshi Yoshioka, Natsuo Yasui, Akira Dezawa

**Affiliations:** 1 Department of Radiological Sciences, University of California, Irvine, CA USA; 2 Department of Orthopedic Surgery, University of California, Irvine, CA USA; 3 Department of Orthopedics, Institute of Health Biosciences, The University of Tokushima Graduate School, Tokushima, Japan; 4 Department of Orthopaedic Surgery, School of Medicine, Teikyo University, Mizonokuchi Hospital, 3-8-3 Mizonokuchi, Takatsu-ku, Kawasaki, 213-8507 Japan

**Keywords:** Laminolysis, Spondylolysis, Retroisthmic cleft, Lumbar spine, Laminar fracture, Stress fracture

## Abstract

Bony defects in the spine are divided into three main types: spondylolysis, pediculolysis, and laminolysis. Lumbar spondylolysis is a well-known stress fracture that occurs frequently in adolescent athletes. Pediculolysis means stress fracture of the pedicle, which sometimes occurs subsequent to unilateral spondylolysis. Laminolysis is a rarely reported stress fracture similar to spondylolysis and pediculolysis that sometimes causes low back pain (LBP). However, its pathomechanism has not been elucidated. Recently, we encountered four adolescent athletes with symptomatic laminolysis. Mean age was 15.8 (range 15–17) years. All subjects reported severe LBP exacerbated by extension of the lumbar spine, and radiology revealed two types of laminolysis: hemilaminar type and intralaminar type. To elucidate the mechanisms of each type, we reviewed a biomechanical study, and found that the hemilaminar type was thought to be subsequent to contralateral spondylolysis, while the intralaminar type might be a result of a stress fracture due to repetitive extension loading.

## Introduction

Bony defects in the posterior elements of the spine are divided into three main types: spondylolysis, pediculolysis, and laminolysis. Lumbar spondylolysis is a well-known stress fracture which occurs frequently in adolescent athletes [1, 2], and is found in around 6 % of the general adult population [3, 4]. Pediculolysis means stress fracture of the pedicle, which sometimes occurs subsequent to unilateral spondylolysis [5]. Laminolysis is a defect in the lamina, similar to spondylolysis and pediculolysis, and is reported to cause low back pain (LBP) in adolescent athletes [6]. Although laminolysis is regarded as a stress fracture [6], its pathomechanism has not been clearly elucidated.

We have previously reported two cases of laminolysis [6]. Here, we present four cases, including the previous two cases, of adolescent athletes with laminolysis. On the basis of radiological and biomechanical findings for the defects, we propose two main types of laminolysis.

## Case reports

Four adolescent athletes (mean age, 15.8 years; range 15–17 years) with laminolysis complained of LBP which prolonged for more than six months. All experienced severe LBP exacerbated by extension of the lumbar spine. For diagnosis, we performed plain radiographs, computed tomography (CT), and magnetic resonance imaging (MRI) for all patients. On the basis of radiological findings, we divided the cases into two types depending on the site of laminolysis: hemilaminar type (cases 1 and 2) and intralaminar type (cases 3 and 4). All patients provided informed consent to participate in the study.

### Hemilaminar type

#### Case 1

**Fig. 1:**
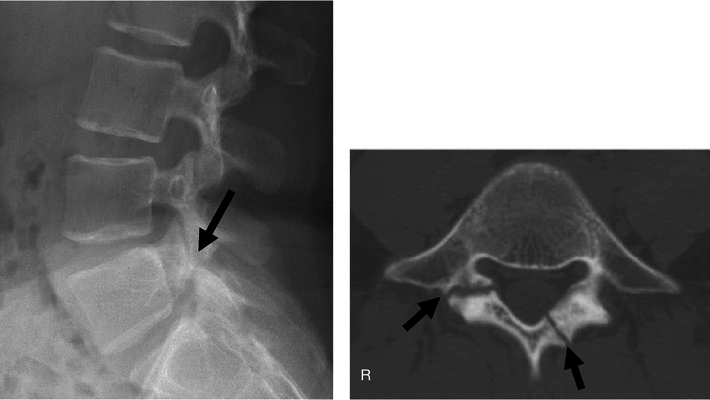
*Left panel*: lateral plain X-ray shows a defect in the L5 lamina. *Right panel*: CT images show two fracture lines. One is seen in the right pars interarticularis (spondylolysis), and the other is seen in the left lamina (laminolysis)

A 17-year-old ballet dancer experienced LBP which prolonged for several months and was exacerbated by extension of the lumbar spine. CT images revealed fractures in the right pars interarticularis (spondylolysis) and the left lamina (laminolysis) (Fig. 1). Therefore, we diagnosed the case as hemilaminar-type laminolysis and spondylolysis. As the LBP was almost resolved, we permitted the patient to return to ballet.

#### Case 2

**Fig. 2:**
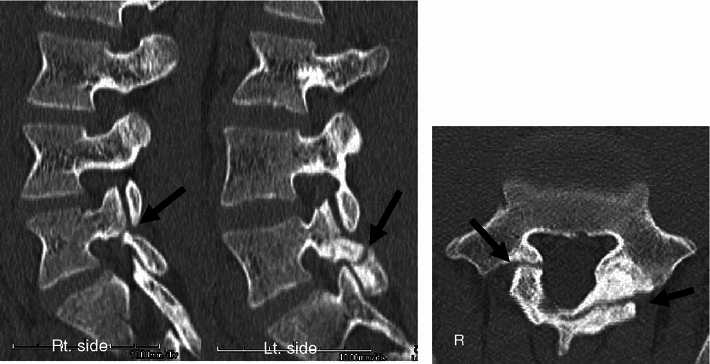
CT images show a pars defect (spondylolysis) on the right side and laminolysis on the left side

A 15-year-old baseball player suffered repeated LBP which was so severe that he experienced sleep disturbances. CT images revealed terminal-stage spondylolysis on the right side and laminolysis on the left side (Fig. 2). Although the patient was treated with conservative treatment such as a hard brace, the LBP did not resolve. Therefore, surgical intervention was performed. Six months later, bony union was obtained and the LBP was resolved, and the patient returned to baseball.

### Intralaminar type

#### Case 3

**Fig. 3:**
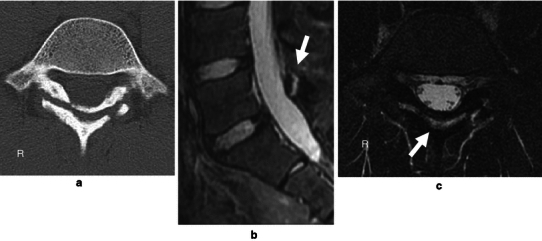
**a** CT images show a clear defect in the L5 lamina, which is seen intralaminally. **b** MRI shows signal changes along the fracture line with high intensity on a fat-saturated T2-weighted image

A 15-year-old Sumo wrestler presented with severe LBP which was exacerbated by extension of the lumbar spine. CT images revealed a clear defect in the L5 lamina which resembled double laminae, suggesting pseudoarthrosis in the lamina (laminolysis) (Fig. 3a). MRI revealed signal changes along the defect of the L5 lamina (Fig. 3b, c). Xylocaine and steroid were injected into the defect. Immediately after the injection, the LBP was completely relieved, and the patient was permitted to resume all activities. At follow-up approximately two years later, he was very active as a professional Sumo wrestler.

#### Case 4

**Fig. 4:**
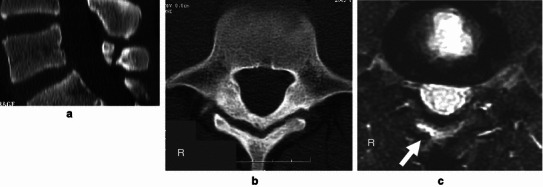
**a** CT images show a bony defect intralaminally. **b** MRI shows signal changes along the defect with high intensity on a fat-saturated T2-weighted image

A 16-year-old boy presented with a three-year history of LBP which was exacerbated by extension of the lumbar spine. CT images revealed sacralization of L5 and the most caudal lumbar vertebra was present at L4, and an apparent defect in the L4 lamina was thought to be bilateral intralaminar laminolysis (Fig. 4a, b). MRI showed signal changes along the defect in the L4 lamina (Fig. 4c). As the LBP on the first presentation was not severe, the patient was treated by immobilization with a soft-trunk brace and allowed to resume all activities. At follow-up one year later, he was very active as a soccer player.

## Discussion

Various types of bony defects in the spine have been reported [1, 4, 5, 7]. They are termed spondylolysis, pediculolysis, and laminolysis according to the defect site. All are regarded as stress fractures, and constitute the main factors of LBP in adolescent sports players. In particular, Micheli and Woods reported that 47 % of adolescent patients with LBP who visited a sports clinic had lumbar spondylolysis [8]. Laminolysis is often described as an incidental finding [9], and sometimes causes LBP in athletes [6].

Here, we found two types of laminolysis: hemilaminar type (cases 1 and 2) and intralaminar type (cases 3 and 4). The former has a cleft in the unilateral pars and the latter has a coronal fracture line though both laminae. In a biomechanical study, Sairyo et al. reported that extension loading causes a more coronally oriented fracture line, while rotational loading causes a more sagittally oriented fracture line [10]. Therefore, the intralaminar type is considered to result from a stress fracture due to repetitive extension loading. In the presence of hemilateral spondylolysis, axial rotation of the contralateral pedicle and pars interarticularis increases stress an average of 6.8-fold, with a 12.6-fold increase being the highest [5].

Patients with spondylolysis are treated conservatively; relative rest, abstinence from sports, and use of a trunk brace. Similar to spondylolysis, conservative treatment is effective for pain relief in patients with laminolysis. From a biomechanical perspective, reducing extension loading should be an effective way to treat intralaminar-type laminolysis. In addition, to prevent hemilaminar-type laminolysis, patients with unilateral spondylolysis need be carefully followed.
